# Echinacoside Alleviates UVB Irradiation-Mediated Skin Damage via Inhibition of Oxidative Stress, DNA Damage, and Apoptosis

**DOI:** 10.1155/2017/6851464

**Published:** 2017-10-26

**Authors:** Di Zhang, Chengtao Lu, Zhe Yu, Xiayin Wang, Li Yan, Juanli Zhang, Hua Li, Jianbo Wang, Aidong Wen

**Affiliations:** ^1^Department of Pharmacy, Xijing Hospital, Fourth Military Medical University, Xi'an 710032, China; ^2^Department of Pharmaceutical Analysis, School of Pharmacy, Fourth Military Medical University, Xi'an 710032, China; ^3^Department of Natural Medicine, School of Pharmacy, Fourth Military Medical University, Xi'an 710032, China; ^4^International Center for Translational Chinese Medicine, Sichuan Academy of Chinese Medicine Sciences, Chengdu 610041, China

## Abstract

Ultraviolet B (UVB) irradiation has been known to cause skin damage, which is associated with oxidative stress, DNA damage, and apoptosis. Echinacoside is a phenylethanoid glycoside isolated from *Herba Cistanches*, which exhibits strong antioxidant activity. In this study, we evaluate the photoprotective effect of echinacoside on UVB-induced skin damage and explore the potential molecular mechanism. BALB/c mice and HaCaT cells were treated with echinacoside before UVB exposure. Histopathological examination was used to evaluate the skin damage. Cell viability, lactate dehydrogenase (LDH) levels, antioxidant enzyme activities, reactive oxygen species (ROS) generation, DNA damage, and apoptosis were measured as well. Western blot was used to measure the expression of related proteins. The results revealed that pretreatment of echinacoside ameliorated the skin injury; attenuated oxidative stress, DNA damage, and apoptosis caused by UVB exposure; and normalized the protein levels of ATR, p53, PIAS3, hnRNP K, PARP, and XPA. To summarize, echinacoside is beneficial in the prevention of UVB-induced DNA damage and apoptosis of the skin *in vivo and in vitro.*

## 1. Introduction

As the outmost layer of the human body, the skin could be seriously damaged when constantly exposed to chemical pollutants and environmental ultraviolet (UV) radiation. UVB radiations (280–320 nm), which are one of the most damaging of the solar UV emissions, could affect various skin structures, cause edema, erythema, hyperplasia, wrinkling, roughness, and premature aging, and even lead to diagnosed skin malignancy [1]. Studies have revealed that chronic exposure of skin to UVB irradiation increases the level of reactive oxygen species (ROS) [2], which resulted in oxidative damage of cellular substrates, such as lipids, proteins, and nucleic acids, leading to inflammation, immunosuppression, apoptosis, and gene mutation [3].

Antioxidant supplementation is an effective strategy to counteract the deleterious effects of ROS generated by superoxide radical, hydroxyl radical, and singlet oxygen after UVB irradiation and reduce the harmful effect of DNA damage by excessive exposure to UVB [4]. Over the past years, there has been a growing interest in the skin care product industry in the use of natural antioxidants to inhibit the adverse effects of UVB or improve the health of irradiated skin. Phenylethanoid glycosides are very protecting promising molecules against ROS' damaging effects, as they share the common structural group phenol, an aromatic ring linkage with at least one hydroxyl substituent. It has been reported that some phenylethanoid glycosides can be applied for photoprotection against UV-induced oxidative stress, owing to the strongest scavenging activity against free radical and their ability to modulate multiple pathways [5, 6].

**Figure 1:**
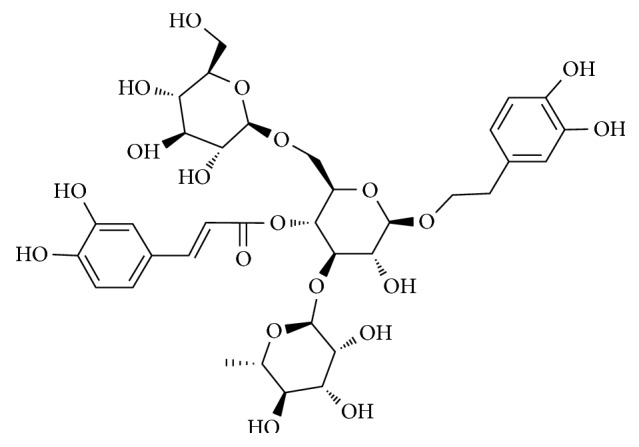
Chemical structure of echinacoside.

Herba Cistanches [7] is one of the most important herbs in traditional Chinese medicine and has been widely used in East Asian countries to treat aging, senescence, and irradiation-related skin disorders. Echinacoside (Figure 1) is one of the major phenylethanoid glycosides isolated and purified from *Cistanches herba* [8]. It is a hydrophilic polyphenol glycoside consisting of a phenylpropanoid and a phenylethanoid glycosidically linked to a trisaccharide moiety, which has strong scavenging activity to scavenge hydroxyl radicals, superoxide anion, and lipid radicals [9] as well as inhibits autoxidation of linoleic acid [10]. In addition, the previous study has revealed that echinacoside and caffeoyl conjugates can prevent the degradation of Type III collagen and protect the skin against oxidative stress induced by UVA/UVB, indicating the potential role of echinacoside for the prevention and treatment of the photodamaged skin [11]. However, information on the critical role of the photoprotective effects of echinacoside against UVB-induced skin damage and its possible mechanism remains limited.

Therefore, this study was undertaken to investigate the protective effect and potential mechanism of echinacoside in UVB-mediated response using mice dorsal epidermal model *in vivo* as well as immortalized human keratinocyte (HaCaT) cells *in vitro*.

## 2. Materials and Methods

### 2.1. Preparation of Echinacoside Formulations

The present study was performed using an o/w cream formulation containing octadecanoic acid, liquid paraffin, and glycerol monostearate as the oil phase and triethanolamine as the main water phase. Echinacoside (Cat number A1019, 99.5%, purity; Mansite Bio-Tech. Co., Ltd., Chengdu, Sichuan, China) was solubilized in distilled water and incorporated into this system. The drug loading capacities of echinacoside in the creams were 0.5%, 1%, and 5%, respectively.

### 2.2. Experimental Animals and UVB Irradiation

Male BALB/c mice (6–8 weeks old) were purchased from the Experimental Animal Research Center, the Fourth Military Medical University (Xi'an, China). The animals were allowed to acclimatize for 1 week and maintained in standard conditions (12 h light/dark cycle, 20.3–23.1°C and 40–50% humidity) during the experimental cycle and fed with standard laboratory food and water ad libitum. All experimental procedures were reviewed and approved by the Institutional Animal Care and Use Committee of the Fourth Military Medical University (protocol number 2014-0415-R).

After shaving, clean the dorsal skins with a hand razor in the tail-to-head direction without damaging the skin. The animals were divided into six groups (*n* = 10): (a) untreated control, (b) UVB, (c) vehicle + UVB, (d) 0.5% echinacoside + UVB, (e) 1% echinacoside + UVB, and (f) 5% echinacoside + UVB.

UVB source used in the experiment was Philips TL 40 W/12 RS (Holland) emitting a continuous spectrum between 270 nm and 400 nm, with a peak emission at 313 nm. Vehicle cream or echinacoside cream was topically applied to the back of each mice. The dose rates of echinacoside were 0.05 mg/cm^2^, 0.1 mg/cm^2^, and 0.5 mg/cm^2^, respectively. After 30 min, mice were exposed to UVB in a special designed cage, and the irradiation intensity was 0.326 mW/cm^2^ as measured by a Sentry ST 513 UV light meter (Taiwan, China). Irradiation dose was calculated using the formula: dose (mJ/cm^2^) = exposure time (sec.) × intensity (mW/cm^2^).

Mice were exposed to UVB every other day ten times with a total energy dose of 1956 mJ/cm^2^ and were sacrificed after 24 h of the last UVB exposure. Histopathology examination and immunohistochemical analysis were performed by placing a part of the dorsal skin in 10% phosphate-buffered formalin. The remainder of the skin tissues was stored in liquid nitrogen.

### 2.3. Skin Edema Analysis

The effect of echinacoside on UVB-induced skin edema was measured as an increase of the dorsal skin weight. After the dorsal skin was excised from the mice, a constant area (5 mm diameter) was delimited using a mold and then weighed.

### 2.4. Hematoxylin and Eosin (H&E) Staining

Five-micrometre-thick sections were obtained from paraffin-embedded tissues for H&E staining. After deparaffinization with xylene and hydration with an alcohol series, the sections were stained with hematoxylin solution (Cat number H6927, Sigma, St. Louis, MO, USA) for 30 s, washed, stained with eosin solution (Cat number 230251, Sigma, St. Louis, MO, USA) for 1 min, washed again, dehydrated with an alcohol series, and cleared with xylene. After mounting, the tissues were observed by Olympus CXX41SF inverted light microscopy (Tokyo, Japan).

### 2.5. Immunohistochemical Detection of Cyclobutane Pyrimidine Dimers (CPDs) and 8-Hydroxy-2′-deoxyguanosine (8-OHdG)

The paraffin-embedded skin sections were deparaffinized, rehydrated, and washed in phosphate-buffered saline (PBS) for CPDs and 8-OHdG detection *in vivo*. The tissue sections were subjected to antigen retrieval by microwaving for 10 min in EDTA antigen retrieval buffer (pH 8.0). Endogenous peroxidase activity was blocked by incubation with 3% H_2_O_2_ for 25 min. After PBS washing, the specimens were incubated in PBS containing 10% goat serum for 30 min to block nonspecific binding sites. Sections were then incubated with mouse monoclonal antibodies against CPDs (Cat number SAB3105004, Sigma, St. Louis, MO, USA) and 8-OHdG (Cat number MOG-100P, clone N45.1, JaICA, Tokyo, Japan). After incubation overnight at 4°C and washed with PBS, the tissues were incubated with biotin-conjugated goat anti-mouse IgG (Cat number A315532, Invitrogen Corporation, CA, USA) for 1 h, with HRP-conjugated streptavidin (Cat number P039701-2, DAKO, Carpienteria, CA) for 30 min and with 3,3′-diaminobenzidine (Cat number K3468, DAKO, Carpienteria, CA) for 5 min. All sections were lightly counterstained with haematoxylin for 2 min followed by dehydration and observed by Olympus CXX41SF inverted light microscopy (Tokyo, Japan).

### 2.6. Antioxidant Enzyme Assays

After the removal of the subcutaneous tissue, the skin tissue fragment was weighed accurately and homogenized with cold Tris-HCl (5 mmol/L, containing 2 mmol/L EDTA, pH 7.4) to prepare 10% (*w*/*v*) tissue homogenate by a polytron homogenizer (Brinkman Kinematica, Switzerland), which was then centrifuged for 15 min at 3000 rpm at 4°C. The supernatant was collected and stored at −80°C. The protein content of each sample was measured by BCA protein assay kit (Cat number P0012, Beyotime Institute of Biotechnology, China).With the respective detection kits (Nanjing Jiancheng Bioengineering Institute, China), the activities of superoxide dismutase (SOD) (Cat number A001), catalase (CAT) (Cat number A007), glutathione peroxidase (GSH-Px) (Cat number A005), and the content of malondialdehyde (MDA) (Cat number A003-1) were determined.

### 2.7. Cell Culture and UVB Irradiation

HaCaT cells (ATCC, Rockville, MD, USA) were cultured in RPMI 1640 medium supplemented with 10% fetal bovine serum (FBS), penicillin (100 U/mL), and streptomycin (100 *μ*g/mL) and incubated at 37°C under 5% CO_2_ in a humidified atmosphere.

The cells were irradiated in culture plates placed under a Philips TL 40W/12 RS UVB lamp (Holland) emitting a continuous spectrum between 270 nm and 400 nm, with a peak emission at 313 nm. The emitted radiation dose was measured by a Sentry ST 513 UV light meter (Taiwan, China).

### 2.8. Determination of Optimal UVB Irradiation Level

The optimum level of UVB irradiation intensity was determined by incubating the cells at a density of 1 × 10^5^ cells/mL in 96-well plates. At a confluence of 80–90%, the cells were exposed to UVB in the range of 25–500 mJ/cm^2^ in 200 *μ*L PBS. After irradiation, the cells were stored in serum-free RPMI 1640 medium for 24 h [12].

### 2.9. Cytotoxicity Determination by 3-(4,5-Dimethylthiazol-2-yl)-2,5-diphenyltetrazolium Bromide (MTT) and Lactate Dehydrogenase (LDH) Assays

Cell proliferation was assessed by MTT assay. Cells were grown in 96-well plates at a density of 1 × 10^5^ cells/mL. At a confluence of 80–90%, cells were pretreated with various concentrations (25, 50, and 100 *μ*mol/L medium) of echinacoside in FBS-free RPMI 1640 medium. After incubation for 24 h, they were washed with PBS and stimulated by UVB irradiation in 200 *μ*L PBS. Subsequently, cells were grown in fresh serum-free medium and incubated for 24 h. The supernatant was removed and 30 *μ*L MTT (5 mg/mL in PBS) was added to each plate and incubated for another 4 h. Then, the supernatant was discarded, and 150 *μ*L of dimethyl sulfoxide was added to dissolve the formazan crystals. The absorbance of each sample was recorded at 490 nm with a Model 680 Microplate Reader (Bio-Rad, Hercules, CA, USA).

Cell injury was measured by quantifying the amount of LDH, a cytosolic enzyme released in the supernatant of cultures by damaged cells. The conditioned media of the UVB-exposed cells were collected for LDH measurement following the supplier's instructions (Cat number A020, Nanjing Jiancheng Bioengineering Institute, China). The absorbance was determined at a wavelength of 450 nm immediately using a Model 680 Microplate Reader (Bio-Rad, Hercules, CA, USA).

### 2.10. Antioxidant Enzyme Assays

After treatment, the cells were washed twice with cold PBS and lysed with RIPA buffer. The protein concentration was determined using the BCA protein assay kit (Beyotime Institute of Biotechnology, China). The activities of SOD (Cat number A001), CAT (Cat number A007) and GSH-Px (Cat number A005), and the cellular content of MDA (Cat number A003-1) were determined following kit's specifications (Nanjing Jiancheng Bioengineering Institute, China).

### 2.11. Determination of Intracellular ROS Production

The level of intracellular ROS generation was detected using 2′, 7′-dichlorofluorescein diacetate (DCFH-DA). HaCaT cells at a concentration of 1 × 10^5^ cells/mL were seeded in 6-well plates. After treatment, DCFH-DA (10 *μ*M) (Cat number D6883, Sigma Chemical Co., St. Louis, MO, USA) was introduced into the cells of each plate, and fluorescence was measured at 488 nm excitation and 525 nm emission using a flow cytometer (Bacton Pickinson, San Jose, CA, USA) and a Fluoview FV1000 Confocal Laser-Scanning Microscope (Olympus, Tokyo, Japan). Fluorescence intensity in cells was analyzed by Olympus FV10-ASW software.

### 2.12. CPD Quantitation In Vitro

UV-induced CPDs were quantitated using the OxiSelect UV-induced DNA damage enzyme-linked immunosorbent assay (ELISA) kit (Cat number SAT-326, Cell Biolabs, Inc., San Diego, CA) according to the manufacturer's instruction.

### 2.13. 8-OHdG Detection In Vitro

HaCaT cells were cultured in sterilized cover slips. At the end, the cells were treated with lysis buffer, and the lysates were centrifuged at 10,000 rpm for 3 min. The supernatants were collected to determine 8-OHdG levels. A standard ELISA procedure was conducted according to the manufacturer's manual (Cat number QS440011, Beijing Gersion Bio-Technology Co., Ltd., China).

### 2.14. DNA Fragmentation Analysis

DNA fragmentation was quantitatively assayed by cytoplasmic histone-associated DNA fragmentation ELISA kit (Cat number 11585045001, Roche, Indianapolis, IN, USA) and agarose gel electrophoresis. HaCaT cells were seeded in 24-well plates and cultured as described above. The cells were collected, and cytoplasmic histone-associated DNA fragmentation was measured according to the manufacturer's instructions.

The pattern of DNA cleavage was analyzed by agarose gel electrophoresis. DNA was extracted by DNA purification kit (Cat number C0007, Beyotime Institute of Biotechnology, Haimen, China) as described by the supplier's instructions. Then the DNA samples mixed with loading buffer [glycerol (40%, *v*/*v*); bromophenol blue (0.25%, *w*/*v*) 2 : 1] and 10 *μ*L were subjected to 1% agarose gel electrophoresis, stained with ethidium bromide (0.5 *μ*g/mL) (Cat number sc-203,735, Santa Cruz Biotechnology Inc., Heidelberg, Germany), and visualized under UV light (the Bio-Rad ChemiDoc XRS imaging system, Hercules, CA, USA).

### 2.15. Fluorescent Terminal Deoxynucleotidyl Transferase (TdT)-Meditated dUTP-Fluorescein Nick End-Labeling (TUNEL) Assay for Detection of Apoptosis

Apoptotic cells were measured using the Cell Death Detection kit (Cat number 11684817910, Roche, Indianapolis, IN, USA). The cells were washed twice with PBS, fixed in 2% paraformaldehyde for 30 min, and permeabilized with 0.1% Triton X-100 for 30 min. They were then incubated with TUNEL reaction buffer at 37°C for 1 h in the dark, rinsed twice with PBS, and incubated with Hoechst 33342 at 37°C for 5 min. The stained cells were visualized under a Nikon TE2000-E inverted fluorescence microscope (Nikon Instruments Inc., Lewisville, TX, USA).

### 2.16. Apoptosis in Flow Cytometry

Annexin V-fluorescein isothiocyanate (FITC)/propidium iodide (PI) double staining was used to measure percentile of apoptosis in HaCaT cells. Cells at a concentration of 1 × 10^5^ cells/mL were seeded in 6-well plates. Finally, the cells were resuspended in 500 *μ*L of 1x binding buffer and mixed with Annexin V-FITC/PI (Cat number APOAF, Sigma Chemical Co., St. Louis, MO, USA). After incubation for 30 min, the cells were measured by Accuri C6 flow cytometry (Accuri, Ann Arbor, MI, USA).

### 2.17. Western Blot

HaCaT cells were seeded in 90 mm dishes. After treatment, cells were washed twice with cold PBS and lysed with RIPA buffer. The lysates were centrifuged at 12,000 rpm for 5 min, and supernatants were collected for gel electrophoresis. Protein concentration of each sample was determined using a BCA protein assay kit (Cat number P0012, Beyotime Institute of Biotechnology, China). Isolated proteins were subjected to SDS-PAGE and transferred to polyvinylidene fluoride (PVDF)/nitrocellulose membranes. The blocked membranes (in 5% nonfat dry milk resolved in PBS containing 0.01% Tween 20, PBST) were then incubated with the primary antibodies against ataxia telangiectasia- and Rad3-related protein kinase (ATR) (Cat number sc-28901), phospho-ATR(Cat number sc-109912), p53(Cat number sc-47698), phospho-p53 (Ser^15^) (Cat number sc-135772), protein inhibitor of activated signal transducer and activator of transcription 3 (PIAS3) (Cat number sc-46682), heterogeneous nuclear ribonucleoprotein K (hnRNP K) (Cat number sc-53620), small ubiquitin-related modifier (SUMO-1) (Cat number sc-5308), cleaved poly ADP-ribose polymerase (PARP) (Cat number sc-56197), xeroderma pigmentosum group A (XPA) (Cat number sc-28353) (1 : 500, Santa Cruz Biotechnology Inc., Heidelberg, Germany), and 8-OHdG (Cat number MOG-100P, 1 : 50, clone N45.1, JaICA, Tokyo, Japan) overnight at 4°C and reacted with HRP-conjugated secondary antibodies (Cat number sc-2789, 1 : 3000, Santa Cruz Biotechnology Inc., Heidelberg, Germany) for 1 h. The immunoreactive bands were detected using the Bio-Rad ChemiDoc XRS imaging system and quantity One software (Bio-Rad, Hercules, CA).

### 2.18. Statistical Analysis

Quantitative values were determined in at least three independent experiments and expressed as the means ± standard deviation (SD). A statistical comparison of different treatment groups was determined by one-way analysis of variance (ANOVA) using GraphPad Prism 5.01 (GraphPad Software, Le Jolla, CA). A *P* value of less than 0.05 was considered as statistically significant.

## 3. Results

### 3.1. Echinacoside Improved UVB-Induced Skin Damage in BALB/c Mice

**Figure 2:**
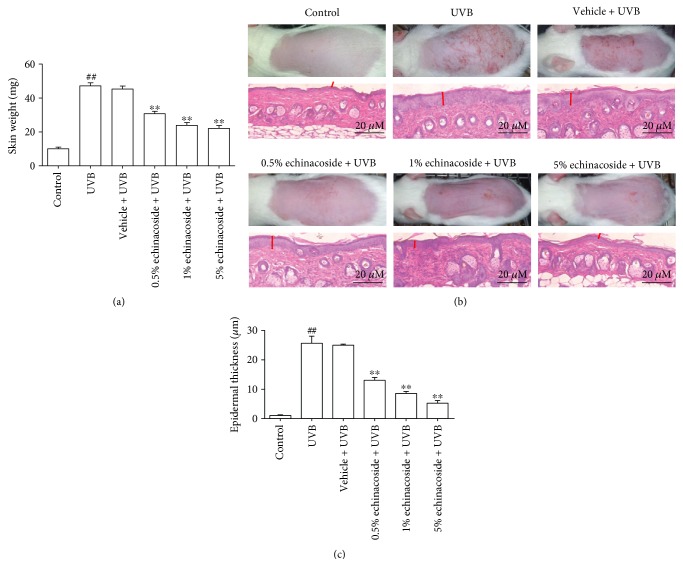
Echinacoside improved UVB-induced skin damage in BALB/c mice. (a) Vehicle or echinacoside cream was administered topically to the back of each mouse. After 30 min, mice were exposed to UVB and exposures were made 10 consecutive days. Animals were sacrificed at 24 h after the last UVB exposure. A constant area (5 mm diameter) was weighed to measure the skin edema. (b) Dorsal skin was photographed. And five-micrometre-thick sections were obtained from paraffin-embedded tissues for H&E staining. The red arrow shows the epidermis thickness of BALB/c mice. (c) Epidermal thicknesses in each group of mice were measured and analyzed. Data are presented as means ± SD (*n* = 10). ^##^*P* < 0.01 versus control group, ^∗∗^*P* < 0.01 versus UVB group.

Compared to unexposed mice, UVB irradiation induced an obvious increase in skin weight (*P* < 0.01). However, the skin weight diminished as the dose of echinacoside increased from 0.5% to 5% (*P* < 0.01) (Figure 2(a)).The result suggested that UVB-induced edema was significantly inhibited by treatment with echinacoside. Based on morphological and histopathological observation of the skin tissues in various groups, we found that exposure to UV resulted in visible skin erythema, wrinkling, and hyperplasia macroscopically. On the contrary, the echinacoside cream partly improved the pathological changes, and the erythema, wrinkling, and hyperplasia were ameliorated with increased concentration of the drug. Micrographs of skin tissues stained with H&E showed that UVB irradiation caused increased thickness (25.62 ± 0.76) of dorsal skins compared with the control group (1.06 ± 0.08) (*P* < 0.01), and the dermal connective tissues were also disorganized. In contrast, the UVB-irradiated mice treated with 0.5%, 1%, and 5% echinacoside showed significantly lower epidermal thickness (13.04 ± 0.30, 8.56 ± 0.21, and 5.25 ± 0.30) (*P* < 0.01), and the dermal morphology appeared to be less damaged than UVB-irradiated or vehicle-treated mice (Figures 2(b) and 2(c)).

### 3.2. Echinacoside Prevented UVB-Induced Cytotoxicity

**Figure 3:**
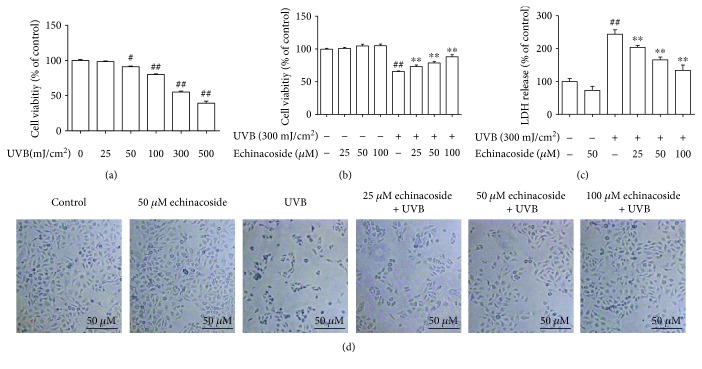
Echinacoside prevented UVB-induced cytotoxicity. (a) Cells were exposed to different doses of UVB (0, 25, 50, 100, 300, and 500 mJ/cm^2^). (b) Cells were pretreated with echinacoside (25, 50, and 100 *μ*M) before exposure to UVB (300 mJ/cm^2^). And 24 h later with UVB irradiation, cell viability was determined by MTT assay. (c) Cell injury was measured by quantifying the amount of LDH in the supernatant of cultures. (d) Morphological changes in HaCaT cells in response to UVB irradiation. Data are presented as means ± SD (*n* = 3). ^#^*P* < 0.05 and ^##^*P* < 0.01 versus control group, ^∗∗^*P* < 0.01 versus UVB group.

We employed MTT and LDH leakage assays to evaluate whether echinacoside prevented UVB-induced cytotoxicity. In MTT assay, UVB radiation at all doses, in the range of 25–500 mJ/cm^2^, both reduced the viability of HaCaT cells, and 300 mJ/cm^2^ UVB irradiation probably induced a 50% decrease in cell viability compared with that in the control group without UVB irradiation (*P* < 0.05 and *P* < 0.01) (Figure 3(a)). Data in Figure 3(b) indicated that treatment of HaCaT cells with echinacoside was not cytotoxic up to 100 *μ*M, whereas the addition of it (25–100 *μ*M) prior to UVB exposure ameliorated UVB-induced cytotoxicity, and this effect was positively correlated with the concentration of the drug (*P* < 0.01). Data from LDH release assay consistently showed that the decrease in the LDH release of cells was detected in the echinacoside-treated group in the presence of UVB irradiation (Figure 3(c)). The inhibitory effect of echinacoside on cell damage by UVB exposure was also confirmed by microscopy. As presented in Figure 3(d), UVB irradiation produced distinct morphological changes in HaCaT cells, causing cell shrinkage and detachment from the cell culture plate, whereas treatment with echinacoside prevented these destructive morphological changes.

### 3.3. Echinacoside Improved the Activities of Antioxidant Enzymes and Inhibited MDA Producing

**Figure 4:**
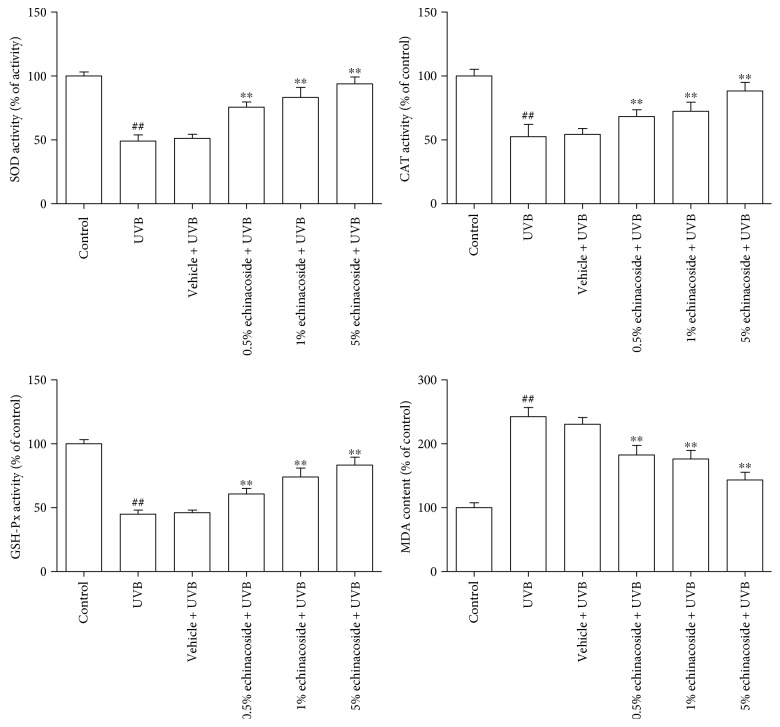
Echinacoside improved the activities of antioxidant enzymes and inhibited MDA production *in vivo*. Vehicle or echinacoside cream was administered topically to the back of each mouse. After 30 min, mice were exposed to UVB and exposures were made 10 consecutive days. Animals were sacrificed at 24 h after the last UVB exposure. The activities of SOD, CAT, and GSH-Px, and the content of MDA of the dorsal skins were measured. Data are presented as means ± SD (*n* = 10). ^##^*P* < 0.01 versus control group, ^∗∗^*P* < 0.01 versus UVB group.

**Figure 5:**
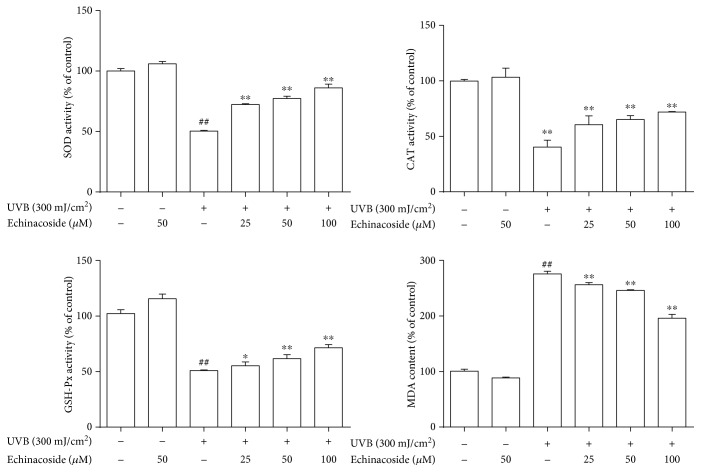
Echinacoside improved the activities of antioxidant enzymes and inhibited MDA production *in vitro*. Cells were pretreated with echinacoside prior UVB exposure. After treatment, the cells were collected to quantify the activities of SOD, CAT, GSH-Px, and the content of MDA. Data are presented as means ± SD (*n* = 3). ^##^*P* < 0.01 versus control group, ^∗^*P* < 0.05 and ^∗∗^*P* < 0.01 versus UVB group.

Data in Figures 4 and 5 suggested that exposure to UVB resulted in a significant reduction in SOD, CAT and GSH-Px activities and an increase in MDA content relative to the control group (*P* < 0.01). However, echinacoside pretreatment improved the activities of SOD, CAT, and GSH-Px and degraded the level of MDA compared with the UVB irradiation group in a concentration-dependent manner both *in vivo* and *in vitro* (*P* < 0.05 and *P* < 0.01).

### 3.4. Echinacoside Suppressed Intracellular ROS Generation

**Figure 6:**
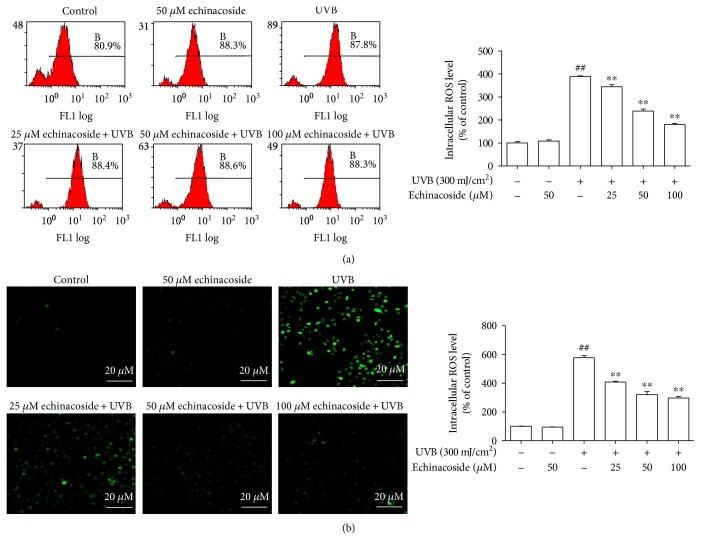
Echinacoside suppressed intracellular ROS generation. (a) Cells were pretreated with echinacoside (25, 50, and 100 *μ*M) before exposure to UVB (300 mJ/cm^2^) irradiation. And 24 h later with UVB irradiation, DCFHDA (10 *μ*M) was introduced into the cells, and fluorescence was measured by flow cytometry. (b) DCFH-DA fluorescence was measured by confocal microscopy. Data are presented as means ± SD (*n* = 3). ^##^*P* < 0.01 versus control group, ^∗∗^*P* < 0.01 versus UVB group.

As an indicator of ROS production, DCFH-DA fluorescence intensity was measured by flow cytometry. Apparently, although the progressive increments in ROS levels were observed in the UVB irradiation group, a significant decrease occurred in HaCaT cells treated with echinacoside (Figure 6(a)). The results were corroborated by morphological observations using confocal microscopy, as expected, pretreated with echinacoside which resulted in lower of ROS level (Figure 6(b)).

### 3.5. Echinacoside Decreased CPD Formation

**Figure 7:**
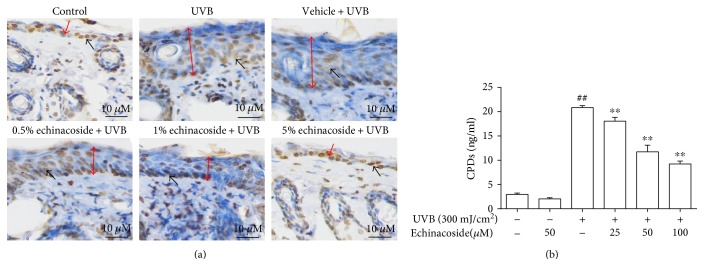
Echinacoside decreased CPD levels in BALB/c mice and HaCaT cells exposed to UVB. (a) Paraffin-embedded sections of the mouse skin were used to immunohistological staining with CPDs. The red arrow shows the epidermis thickness of BALB/c mice, and the black arrow shows CPD-positive cell. (b) Cells were pretreated with echinacoside (25, 50, and 100 *μ*M) prior to UVB exposure. After 24 h, the cells were collected to quantify CPD production using ELISA analysis. Data are presented as means ± SD (*n* = 3). ^##^*P* < 0.01 versus control group, ^∗∗^*P* < 0.01 versus UVB group.

Our result showed strong and intensive staining for CPDs in mouse skin after UVB irradiation. Whereas, comparatively lower intensity of CPDs was easily detectable in the epidermis and dermis following topical treatment of echinacoside (Figure 7(a)). This protective effect of echinacoside against UVB-induced CPD formation was confirmed and further quantified *in vitro.* As shown in Figure 7(b), pretreatment of the cell with echinacoside dose dependently reduced the amount of UVB-generated CPDs.

### 3.6. Echinacoside Reduced the Level of 8-OHdG

**Figure 8:**
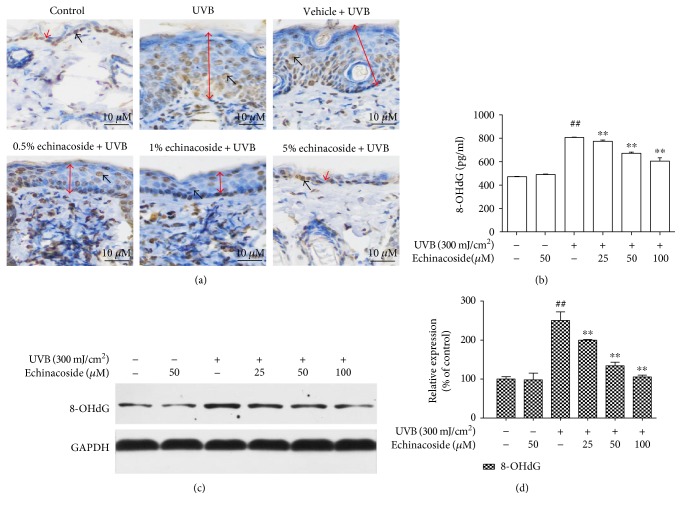
Echinacoside reduced the level of 8-OHdG in BALB/c mice and HaCaT cells exposed to UVB. (a) Paraffin-embedded sections of the mouse skin were used to immunohistological staining with 8-OHdG. The red arrow shows the epidermis thickness of BALB/c mice, and the black arrow shows 8-OHdG-positive cell. (b) Cells were pretreated with echinacoside (25, 50, and 100 *μ*M) prior UVB exposure. After 24 h, the cells were collected to quantify 8-OHdG production using ELISA analysis. (c and d) Protein levels of 8-OHdG in cells were determined by Western blot analysis. Data are presented as means ± SD (*n* = 3). ^##^*P* < 0.01 versus control group, ^∗∗^*P* < 0.01 versus UVB group.

Immunohistochemical staining revealed that in the UVB group, a representative 8-OHdG was localized mainly between the epidermis and dermis. In comparison, a significant inhibition in 8-OHdG induction was observed following topical application of echinacoside (Figure 8(a)). This result was consistent with that of the ELISA test *in vitro*, which showed that the level of 8-OHdG in UVB irradiation cells apparently enhanced compared with the non-UVB-irradiated cells (*P* < 0.01), while it diminished as the echinacoside concentration increased from 25 to 100 *μ*M (Figure 8(b)). A similar result was observed in 8-OHdG expression by Western blot (Figures 8(c)).

### 3.7. Echinacoside Inhibited UVB-Induced DNA Fragmentation

**Figure 9:**
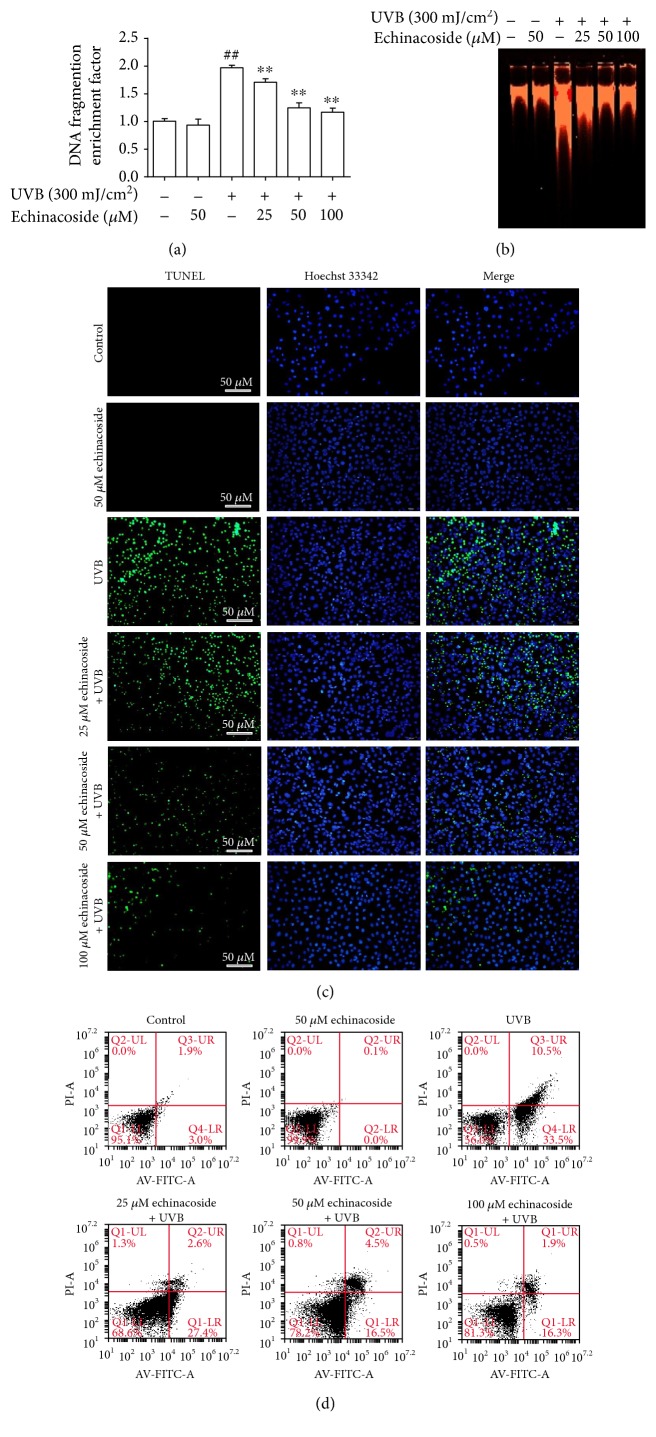
Echinacoside inhibited UVB-induced DNA fragmentation and apoptosis in HaCaT cells. (a) Cells were pretreated with echinacoside (25, 50, and 100 *μ*M) prior UVB exposure. And 24 h later with UVB irradiation, the cells were collected to quantify DNA fragmentation using ELISA analysis. (b) Genomic DNA was isolated and checked for fragmentation on a 1% agarose gel. (c) Cells were treated with echinacoside (25, 50, and 100 *μ*M) and exposed to UVB radiation. Apoptotic bodies were observed by TUNEL assay. (d) The apoptotic cells (annexin V+/PI-) were analyzed by flow cytometer. Data are presented as means ± SD (*n* = 3). ^##^*P* < 0.01 versus control group, ^∗∗^*P* < 0.01 versus UVB group.

UVB irradiation resulted in a marked and significant increase in histone-associated DNA fragmentation compared with the non-UVB group (*P* < 0.01). However, the fragmentation in echinacoside-pretreated cells was significantly lower than that in the UVB-exposed cells (*P* < 0.01), and this effect was more prominent in higher concentrations (Figure 9(a)). Following agarose gel electrophoresis, UVB irradiation produced a typical ladder with clearly increased intensity of DNA fragmentation, and the application of echinacoside before UVB exposure significantly reduced its formation (Figure 9(b)).

### 3.8. Echinacoside Blocked UVB-Induced Apoptosis

As illustrated, there were a large number of TUNEL-positive cells in the UVB-irradiated group compared with very few apoptotic cells in the control group. However, after pretreating with 25, 50, or 100 *μ*M echinacoside, the number of TUNEL-positive cells decreased (Figure 9(c)). In addition, the protective effect of echinacoside against apoptosis was also confirmed by flow cytometric quantitative analysis. Exposure of cells with UVB resulted in significant induction of apoptosis (44.0%) compared to non-UVB-exposed cells (4.9%, *P* < 0.01). Treatment with echinacoside at the doses of 25, 50, and 100 *μ*M resulted in a significant reduction in the number of apoptotic cells at both the early and late stages of apoptosis to 30%, 21%, and 18.2% respectively (*P* < 0.01) (Figure 9(d)).

### 3.9. Echinacoside Modulated the Expression of ATR, p53, PIAS3, hnRNP K, PARP, and XPA in HaCaT Cells

**Figure 10:**
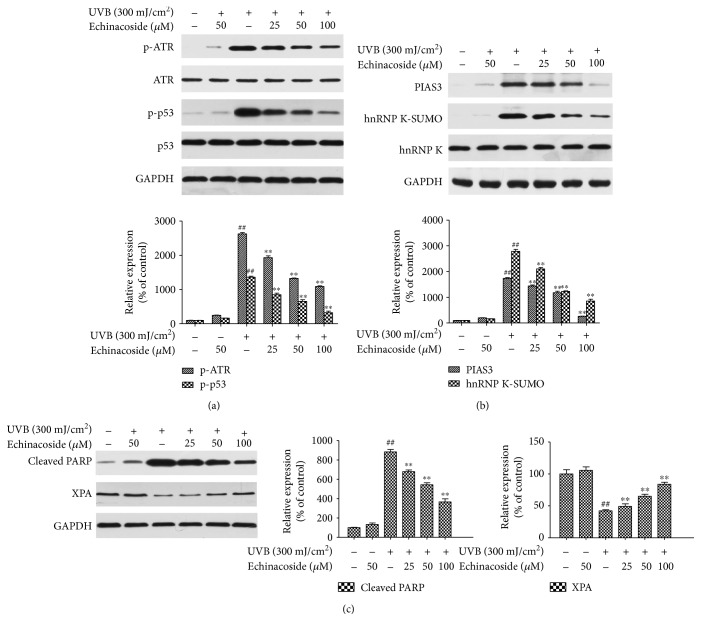
Echinacoside modulated the expression of ATR, p53, hnRNP K, PIAS3, PARP, and XPA in HaCaT cells. (a) Cells were pretreated with echinacoside (25, 50, and 100 *μ*M) prior to UVB exposure. And 24 h later with UVB irradiation, total cell lysates were harvested. The expression of p-ATR, ATR, p-p53, and p53 proteins was determined by Western blotting. (b) The expression of PIAS3, SUMOylated hnRNP K, and hnRNP K proteins was determined by Western blotting. (c) The expression of cleaved PARP and XPA proteins was determined by Western blotting. GAPDH was used as an internal standard. Data are presented as means ± SD (*n* = 3). ^##^*P* < 0.01 versus control group, ^∗∗^*P* < 0.01 versus UVB group.

As shown in Figure 10(a), UVB induced the phosphorylation of ATR and p53 (Ser^15^). Whereas, the administration of echinacoside (25, 50, or 100 *μ*M) before UVB exposure markedly inhibited the increase in the levels of phospho-ATR and phospho- p53 (Ser^15^) and had little or no effect on them in the absence of UVB. Cell exposure to UVB exhibited higher levels of PIAS3 and SUMOylated hnRNP K protein, whereas treatment with echinacoside was associated with a dose-dependent decrease in the expression of PIAS3 and SUMOylated hnRNP K (Figure 10(b)). In HaCaT cells, UVB irradiation caused an increase in cleaved PARP and pretreatment with echinacoside inhibited the activation of cleaved PARP. Pretreatment with echinacoside restored the reduced XPA expression caused by UVB as well (Figure 10(c)).

## 4. Discussion

Studies have revealed that the photodamaged skin involves increased epidermal thickness and alterations in connective tissue organization [13]. Similarly, in the present study, chronic UVB irradiation induced edema, erythema, wrinkling, and epidermal thickening in the mouse skin. Interestingly, we found that echinacoside could inhibit UVB and caused mouse skin damage in a dose-dependent manner.

Evidences have been shown that UVB radiation produced DNA damage directly and indirectly through oxidative stress in human skin and also the induction of apoptosis as a protective mechanism relevant in limiting the survival of cells with irreparable DNA damage caused by UVB [14, 15]. Previous studies have indicated that echinacoside could reduce ROS accumulation, alleviate DNA damage, and protect cells against apoptosis [16–18]. In this study, we also found that echinacoside displayed a protective effect against UVB-induced oxidative stress and DNA damage in the skin with a concomitant decrease in the apoptotic response.

Oxidative stress plays a significant role in UVB-induced skin damage [19]. UV irradiation produces ROS, causing the depletion of the cellular antioxidant defense system [20]. ROS-derived free radicals can react with lipids, proteins, and DNA, if not blocked by sufficient antioxidant molecules, to form lipid peroxides which can lead to extensive cell damage and death [21]. Overproduction of ROS may be responsible for the observed membrane damage as evidenced by the elevated lipid peroxidation in terms of MDA in the present research. Whereas, levels of ROS and MDA were both remarkably downregulated by echinacoside. In addition, free radical scavenging enzymes, such as SOD, CAT, and GSH-Px, are the first line of cellular defense against oxidative injury, decomposing O_2_, and H_2_O_2_ before their interaction to form the more reactive hydroxyl radical. The equilibrium between the enzymatic antioxidants and free radicals is an important process for the effective removal of oxidative stress in intracellular organelles. In this study, a significantly lower activity of the enzymes SOD, CAT, and GSH-Px was observed *in vivo* and *in vitro* after UVB irradiation, which is consistent with similar findings in a number of earlier studies [22, 23]. However, we found that echinacoside markedly increased SOD, CAT, and GSH-Px activities in UVB-irradiated mice dorsal skin and HaCaT cells (Figures 4 and 5). Our finding suggests that echinacoside could attenuate oxidative stress by decreasing the levels of ROS and lipid peroxide, thus resulting in the enhancement of the antioxidant system of the mice dorsal skin and HaCaT cells irradiated by UVB.

UVB is strongly absorbed by cellular DNA in the skin and results in several different types of premutagenic lesions, which could alter the structure of DNA and consequently inhibit polymerases and arrest replication. CPDs are common photochemical products with respect to photocarcinogenesis [24, 25]. CPDs formed more abundantly and repaired less efficiently than any other photoproducts, thus are considered to be the predominant UVB-induced DNA photolesions [26, 27]. 8-OHdG, physiologically formed and enhanced by chemical and physical carcinogens, is also a ubiquitous marker of oxidative stress among numerous types of oxidative DNA damage. In the present study, we demonstrated that echinacoside inhibited CPDs and 8-OHdG production and reduced the formation of DNA fragmentation induced by UVB.

Phosphoinositol-3-phosphate kinase-like kinase ATR is critical to the proper function of DNA damage and can be activated in response to a variety of damaging agents, particularly to the UV irradiation damage [28]. It is well established that ATR kinase mediated the activation of tumor suppressor p53, a critical gene in the regulation of cell arrest, DNA repair, and apoptosis. ATR phosphorylates p53 at Ser^15^, increasing its transactivation activity [29, 30] and thus inducing the expression of a variety of downstream genes, leading to apoptosis [31]. In the present study, our results are in agreement with Tibbetts et al.'s [32] previous work, in which UV irradiation induced the phosphorylation of ATR and p53 (Ser^15^). Significantly, the levels of phosphorylated ATR and p53 were further suppressed by echinacoside treatment.

As a component of the hnRNP complex, hnRNP K plays an essential role in RNA and DNA binding [33]. Specifically, following its ATR-dependent induction after UV irradiation, hnRNP K will activate p53 target genes in cooperation with p53 and thereby lead to the trigger of cell-cycle-checkpoint events [34]. SUMO is an ubiquitin-like protein that is conjugated to a variety of cellular proteins. Like ubiquitin, SUMO is conjugated to target proteins by a cascade enzyme system consisting of E1 activating enzyme, E2 conjugating enzyme, and PIASs. The previous study has found that UV-induced SUMOylation of hnRNP K is mediated by PIAS3, and the SUMOylation can increase stability of hnRNP K in an ATR-dependent manner, leading to cell-cycle arrest [35]. In the current study, we found that PIAS3 ligated SUMO to hnRNP K upon exposure to UVB, and echinacoside exerted its resistance function in hnRNP K SUMOylation.

It has been reported that apoptosis could be an oxidative response which is closely associated with DNA damage, and changes in UV-induced apoptosis may have a profound impact in the induction of skin damage. PARP appears to be involved in DNA repair in response to environmental pressure. Cleavage of PARP facilitates cellular disassembly and serves as a marker of cells undergoing apoptosis [36, 37]. The results of Western blots were consistent with the apoptosis analysis of TUNEL and flow cytometry, which showed that echinacoside treatment downregulated the expression of cleaved PARP and blocked UVB-induced apoptosis.

Additionally, XPA deficiency is known to decrease antioxidant defense and increase susceptibility to UVB-induced skin cancer [38]. ATR actively targeted XPA for regulation of its nuclear import in response to DNA damage [39], and p53 also mediates XPA nuclear localization [40]. In the present study, acute exposure of UVB caused a sharp decrease of XPA expression, suggesting that the cells were unable to process the removal of a wide spectrum of DNA lesions under acute external stress. Fortunately, echinacoside restored the expression of XPA, confirming the effect of echinacoside against UVB-induced cell damage by inhibiting DNA damage and promoting repair.

**Figure 11:**
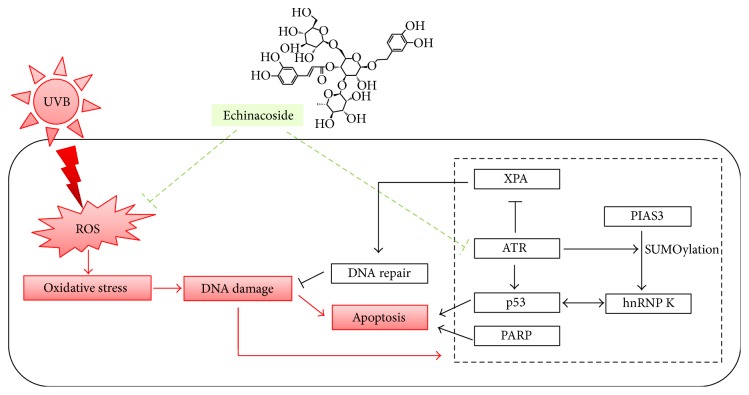
A proposed working model related to potential signaling that is involved in the protective effect of echinacoside against UVB-induced skin damage.

A proposed working model related to the protective effect of echinacoside against UVB irradiation-mediated skin damage was described in Figure 11.

## 5. Conclusion and Perspective

In conclusion, the present study demonstrated that echinacoside manifested significant protective effects against UVB-induced DNA damage and apoptosis. This effect could be at least partly mediated by its antioxidation as well as suppressing the activation of ATR and the downstream genes. This finding might provide evidence supporting the beneficial effects of echinacoside in the prevention and treatment of UVB-associated skin diseases. However, studies are still needed to discriminate the crosstalk among different signaling involved in the protective effect of echinacoside, especially including the use of ATR knockout mice and specific inhibitors. And also, whether posttreatment of echinacoside would be effective for protecting the skin against UVB radiation as well as the effect of echinacoside to malignant cells or UVA/UVC-related skin injury will be identified in further investigations.

## Abbreviations

UVB: Ultraviolet B
HaCaT: Immortalized human keratinocyte
H&E: Hematoxylin and eosin
CPDs: Cyclobutane pyrimidine dimers
8-OHdG: 8-Hydroxy-2′-deoxyguanosine
PBS: Phosphate-buffered saline
SOD: Superoxide dismutase
CAT: Catalase
GSH-Px: Glutathione peroxidase
MDA: Malondialdehyde
MTT: 3-(4,5-dimethylthiazol-2-yl)-2,5-diphenyltetrazolium bromide
LDH: Lactate dehydrogenase
DCFH-DA: 2′, 7′-dichlorofluorescein diacetate
TUNEL: Fluorescent terminal deoxynucleotidyl transferase (TdT)-mediated dUTP-fluorescein nick end-labeling
ATR: Ataxia telangiectasia- and Rad3-related protein kinase
PIAS3: Protein inhibitor of activated signal transducer and activator of transcription 3
hnRNP K: Heterogeneous nuclear ribonucleoprotein K
SUMO-1: Small ubiquitin-related modifier
PARP: Poly ADP-ribose polymerase
XPA: Xeroderma pigmentosum group A
FITC: Annexin V-fluorescein isothiocyanate
PI: Propidium iodide.

## References

[1] Tebbe B. (2001). Relevance of oral supplementation with antioxidants for prevention and treatment of skin disorders. *Skin Pharmacology and Applied Skin Physiology*.

[2] Yin Y., Li W., Son Y. O. (2013). Quercitrin protects skin from UVB-induced oxidative damage. *Toxicology and Applied Pharmacology*.

[3] Lee C. H., Wu S. B., Hong C. H., Yu H. S., Wei Y. H. (2013). Molecular mechanisms of UV-induced apoptosis and its effects on skin residential cells: the implication in UV-based phototherapy. *International Journal of Molecular Sciences*.

[4] Ahmad N., Mukhtar H. (2001). Cutaneous photochemoprotection by green tea: a brief review. *Skin Pharmacology and Applied Skin Physiology*.

[5] Dell'Acqua G., Schweikert K. (2012). Skin benefits of a myconoside-rich extract from resurrection plant *Haberlea rhodopensis*. *International Journal of Cosmetic Science*.

[6] Yang J. H., Meng X. Y., Hu J. P. (2011). Preparation and quality evaluation of congrong whitening sunscreen cream. *West China Journal of Pharmaceutical Sciences*.

[7] Zhao Q., Gao J., Li W., Cai D. (2010). Neurotrophic and neurorescue effects of echinacoside in the subacute MPTP mouse model of Parkinson’s disease. *Brain Research*.

[8] Jia C., Shi H., Wu X., Li Y., Chen J., Tu P. (2006). Determination of echinacoside in rat serum by reversed-phase high-performance liquid chromatography with ultraviolet detection and its application to pharmacokinetics and bioavailability. *Journal of Chromatography. B, Analytical Technologies in the Biomedical and Life Sciences*.

[9] Li J., Zheng R. L., Liu Z. M., Jia Z. J. (1992). Scavenging effects of phenylpropanoid glycosides on superoxide and its antioxidation effect. *Zhongguo Yao Li Xue Bao*.

[10] Zheng R. L., Wang P. F., Li J., Liu Z. M., Jia Z. J. (1993). Inhibition of the autoxidation of linoleic acid by phenylpropanoid glycosides from *Pedicularis* in micelles. *Chemistry and Physics of Lipids*.

[11] Facino R. M., Carini M., Aldini G., Saibene L., Pietta P., Mauri P. (1995). Echinacoside and caffeoyl conjugates protect collagen from free radical-induced degradation: a potential use of Echinacea extracts in the prevention of skin photodamage. *Planta Medica*.

[12] Wagener S., Volker T., De Spirt S., Ernst H., Stahl W. (2012). 3,3′-Dihydroxyisorenieratene and isorenieratene prevent UV-induced DNA damage in human skin fibroblasts. *Free Radical Biology & Medicine*.

[13] Smith J. G., Davidson E. A., Sams W. M., Clark R. D. (1962). Alterations in human dermal connective tissue with age and chronic sun damage. *The Journal of Investigative Dermatology*.

[14] Ichihashi M., Ueda M., Budiyanto A. (2003). UV-induced skin damage. *Toxicology*.

[15] Katiyar S. K., Mantena S. K., Meeran S. M. (2011). Silymarin protects epidermal keratinocytes from ultraviolet radiation-induced apoptosis and DNA damage by nucleotide excision repair mechanism. *PLoS One*.

[16] Deng M., Zhao J. Y., Tu P. F., Jiang Y., Li Z. B., Wang Y. H. (2004). Echinacoside rescues the SHSY5Y neuronal cells from TNFα-induced apoptosis. *European Journal of Pharmacology*.

[17] Geng X., Song L., Pu X., Tu P. (2004). Neuroprotective effects of phenylethanoid glycosides from *Cistanches salsa* against 1-methyl-4-phenyl-1,2,3,6-tetrahydropyridine (MPTP)-induced dopaminergic toxicity in C57 mice. *Biological & Pharmaceutical Bulletin*.

[18] Xie H., Zhu H., Cheng C., Liang Y., Wang Z. (2009). Echinacoside retards cellular senescence of human fibroblastic cells MRC-5. *Pharmazie*.

[19] Halliday G. M. (2005). Inflammation, gene mutation and photoimmunosuppression in response to UVR-induced oxidative damage contributes to photocarcinogenesis. *Mutation Research*.

[20] Petrova A., Davids L. M., Rautenbach F., Marnewick J. L. (2011). Photoprotection by honeybush extracts, hesperidin and mangiferin against UVB-induced skin damage in SKH-1 mice. *Journal of Photochemistry and Photobiology B*.

[21] Melnikova V. O., Ananthaswamy H. N. (2005). Cellular and molecular events leading to the development of skin cancer. *Mutation Research*.

[22] Kim K. C., Piao M. J., Cho S. J., Lee N. H., Hyun J. W. (2012). Phloroglucinol protects human keratinocytes from ultraviolet B radiation by attenuating oxidative stress. *Photodermatology, Photoimmunology & Photomedicine*.

[23] Filip A., Daicoviciu D., Clichici S. (2011). The effects of grape seeds polyphenols on SKH-1 mouse skin irradiated with multiple doses of UV-B. *Journal of Photochemistry and Photobiology B*.

[24] Protic-Sabljic M., Tuteja N., Munson P. J., Hauser J., Kraemer K. H., Dixon K. (1986). UV light-induced cyclobutane pyrimidine dimers are mutagenic in mammalian cells. *Molecular and Cellular Biology*.

[25] Mitchell D. L., Nairn R. S. (1989). The biology of the (6–4) photoproduct. *Photochemistry and Photobiology*.

[26] Ravanat J. L., Douki T., Cadet J. (2001). Direct and indirect effects of UV radiation on DNA and its components. *Journal of Photochemistry and Photobiology B*.

[27] Vink A. A., Henegouwen B., Nikaido O., Baan R. A., Roza L. (1994). Removal of UV-induced DNA lesions in mouse epidermis soon after irradiation. *Journal of Photochemistry and Photobiology B*.

[28] Cliby W. A., Roberts C. J., Cimprich K. A. (1998). Overexpression of a kinase-inactive ATR protein causes sensitivity to DNA-damaging agents and defects in cell cycle checkpoints. *The EMBO Journal*.

[29] Canman C. E., Lim D. S., Cimprich K. A. (1998). Activation of the ATM kinase by ionizing radiation and phosphorylation of p53. *Science*.

[30] Guo Z., Kumagai A., Wang S. X., Dunphy W. G. (2000). Requirement for Atr in phosphorylation of Chk1 and cell cycle regulation in response to DNA replication blocks and UV-damaged DNA in *Xenopus* egg extracts. *Genes & Development*.

[31] Vogelstein B., Lane D., Levine A. J. (2000). Surfing the p53 network. *Nature*.

[32] Tibbetts R. S., Brumbaugh K. M., Williams J. M. (1999). A role for ATR in the DNA damage-induced phosphorylation of p53. *Genes & Development*.

[33] Zhou R., Shanas R., Nelson M. A., Bhattacharyya A., Shi J. (2010). Increased expression of the heterogeneous nuclear ribonucleoprotein K in pancreatic cancer and its association with the mutant p53. *International Journal of Cancer*.

[34] Moumen A., Masterson P., O'Connor M. J., Jackson S. P. (2005). hnRNP K: an HDM2 target and transcriptional coactivator of p53 in response to DNA damage. *Cell*.

[35] Lee S. W., Lee M. H., Park J. H. (2012). SUMOylation of hnRNP-K is required for p53-mediated cell-cycle arrest in response to DNA damage. *The EMBO Journal*.

[36] Satoh M. S., Lindahl T. (1992). Role of poly(ADP-ribose) formation in DNA repair. *Nature*.

[37] Oliver F. J., de la Rubia G., Rolli V., Ruiz-Ruiz M. C., de Murcia G., Murcia J. M. (1998). Importance of poly(ADP-ribose) polymerase and its cleavage in apoptosis. *The Journal of Biological Chemistry*.

[38] de Vries A., van Oostrom C. T. M., Hofhuis F. M. (1995). Increased susceptibility to ultraviolet-B and carcinogens of mice lacking the DNA excision repair gene XPA. *Nature*.

[39] Wu X., Shell S. M., Liu Y., Zou Y. (2007). ATR-dependent checkpoint modulates XPA nuclear import in response to UV irradiation. *Oncogene*.

[40] Li Z., Musich P. R., Zou Y. (2011). Differential DNA damage responses in p53 proficient and deficient cells: cisplatin-induced nuclear import of XPA is independent of ATR checkpoint in p53-deficient lung cancer cells. *International Journal of Biochemistry and Molecular Biology*.

